# A personalized diet intervention improves depression symptoms and changes microbiota and metabolite profiles among community-dwelling older adults

**DOI:** 10.3389/fnut.2023.1234549

**Published:** 2023-09-14

**Authors:** Faiga Magzal, Silvia Turroni, Marco Fabbrini, Monica Barone, Adi Vitman Schorr, Ariella Ofran, Snait Tamir

**Affiliations:** ^1^Laboratory of Human Health and Nutrition Sciences, MIGAL-Galilee Research Institute, Kiryat Shmona, Israel; ^2^Department of Nutrition, Tel Hai College, Upper Galilee, Israel; ^3^Unit of Microbiome Science and Biotechnology, Department of Pharmacy and Biotechnology, University of Bologna, Bologna, Italy; ^4^Microbiomics Unit, Department of Medical and Surgical Sciences, University of Bologna, Bologna, Italy; ^5^Clalit Health Services, Tel Aviv, Israel

**Keywords:** older adults, depression, microbiome, personalized diet, metabolites, quality of life

## Abstract

**Introduction:**

The impact of diet on mental well-being and gut microorganisms in humans is well recognized. However, research on the connections between food nutrients, gut microbiota, and mental health remains limited. To address this, the present study aimed to assess the effects of a personalized diet, based on individual needs and aligned with the Mediterranean diet principles, on depression symptoms, quality of life, nutritional intake, and gut microbiota changes among older adults living in the community.

**Methods:**

The intervention involved regular visits from a registered dietitian, who provided tailored dietary recommendations. During the 6-month study, participants completed questionnaires to evaluate their depression levels, quality of life, and dietary habits. Additionally, they provided stool samples for analysis of gut microbiota and metabolites.

**Results:**

The results demonstrated that the personalized dietary intervention reduced depression symptoms and improved the quality of life among older adults. Furthermore, significant changes in the intake of certain nutrients, such as folate, lutein, zeaxanthin, EPA, and DHA, were observed following the intervention. Moreover, the intervention was associated with increased diversity in the gut microbiome and reduced total short-chain fatty acids, the main metabolites produced by gut microorganisms. The study also revealed correlations between food nutrients, gut microbiota, and mental health parameters.

**Discussion:**

In conclusion, this research highlights the potential advantages of personalized dietary interventions in managing depression and enhancing overall well-being among older populations. It also sheds light on the role of gut microbiota and its metabolites in these effects. The findings offer valuable insights into the significance of nutrition and gut health for mental well-being in older adults.

## Introduction

Depression refers to various mental problems characterized by low mood and by loss of interest and enjoyment in ordinary experiences. Depression is associated with emotional, cognitive, physical, and behavioral symptoms, which significantly impact functioning and quality of life (1). Depression affects 5.7% of adults older than 60 years, and is foreseen to become the leading contributor to the global disease burden, mainly due to the high societal costs and effects on public health (2).

Various diets and dietary patterns have been implicated in biological mechanisms behind depression; these mechanisms include inflammation, oxidative stress, and tryptophan-kynurenine metabolism (3). Conversely, a high-quality diet may help regulate the gut microbiota, reduce stress and inflammation in the brain, and maintain proper cognitive function throughout life (4). The brain’s composition, structure, and function depend on the availability of appropriate nutrients, including lipids, amino acids, vitamins, and minerals. Thus, diet is a modifiable variable for targeting mental health, mood, and cognitive performance (5, 6).

Intervention studies support the use of adjunctive dietary interventions in improving clinical depression and depressive symptoms. A meta-analysis of primarily non-clinical populations showed that nutritional interventions were followed by modest reductions in depressive symptoms (7). A number of randomized controlled trials in adults showed consistently moderate-to-large improvements in symptoms from Mediterranean diet-based interventions compared to control conditions (8–11). The Mediterranean diet (MD) is characterized by fresh, seasonal, unprocessed food; olive oil; low-fat dairy products; fish and lean chicken parts, and alcohol in moderation. This compares with Western dietary patterns of processed food, red meat, trans fat, excessive alcohol, and few fruits and vegetables. Compared to Western dietary patterns, the Mediterranean diet was shown to be associated with reduced risk of depression in both cross-sectional and prospective studies (8, 12–14).

The gut microbiota has been considered a key variable that links diet and depression (15, 16). Yet, the directionality of these relations still needs to be discerned. Microorganisms present in the gut communicate with the brain through the gut-brain axis network, and thereby influence emotional behavior and neurological processes (17–20). Metabolites, such as short-chain fatty acids (SCFAs), tryptophan derivatives, and secondary bile acids synthesized from dietary components and secreted by gut microbes, can reach and affect the brain by various signaling mechanisms. Similarly, the brain can modulate the microbiota directly by affecting neuroactive substances released into the gut lumen, or indirectly via alterations of the gut microbial environment and behavior (17).

Despite the above, human studies linking the microbiota to diet and mood are scarce (21, 22). For example, Taylor et al. showed associations between bacterial taxa and Depression Anxiety and Stress Scale scores in adults without diagnosed mood disorders (21). Furthermore, an inverse association was reported of total fruit and dairy components with the Depression Anxiety and Stress Scale (23). Following adherence to the Mediterranean diet, older adults were shown to have increased abundance of specific microbiota taxa that were positively associated with several markers of lower frailty and improved cognitive function, and negatively related to inflammatory markers (24).

The objectives of this pre-post study were to examine depression symptoms, quality of life, food nutrient quantities, and SCFA concentrations in community-dwelling older adults after a 6-month personalized intervention based on the principles of the Mediterranean diet. Moreover, we aimed to investigate the interrelations between food nutrients, gut microbiota, and mental health.

## Materials and methods

This study was approved by the ethic committee of the Meir Medical Center (approval no 0186-16-COM1) and was registered at https://clinicaltrials.gov (registration no NCT03256929). All the participants signed an informed consent form, and all study procedures were carried out in accordance with Good Clinical Practice.

### Participants

Announcements calling for volunteers to participate in a dietary intervention were placed in community centers for older adults. The stated eligibility criteria were continuous low mood or sadness, loss of interest, or hopeless and helpless feelings. Additional criteria were age 60 years or older, community-dwelling, good general health, and a Geriatric Depression Scale (GDS) score equal to or above 2. Exclusion criteria were clinically diagnosed and medically treated depression, other severe medical health conditions, such as cancer and inflammatory bowel disease, and the use of antibiotics for at least 4 weeks before enrollment.

Suitable participants underwent a clinical interview conducted by a doctor, during which they were asked about their medical conditions, medications, and other substance use. Anthropometric measurements, depression, and food frequency and quality of life questionnaires were administered, and participants’ fecal samples were collected at baseline (T0). The questionnaires and fecal sample collection were performed again, after 6 months of nutritional intervention (T1).

The reasons for not having a control group in this study were mainly ethical and feasible considerations. We considered it unethical to deny participants access to a potentially beneficial diet intervention since there is strong evidence supporting the positive health effects of the Mediterranean diet (25). Furthermore, we performed a pilot study before starting the research, which highlighted that the participants eventually knew each other since the recruitment was done in community centers in the northern region of Israel and would drop-out when allocated to the control group.

### Study intervention

At home visits, scheduled every 2 weeks during the intervention period, registered dietitians performed a 24-h dietary recall, provided dietary recommendations based on the participants’ personalized health needs and following the Mediterranean-diet pattern. The diet encouraged the consumption of fruits, vegetables, whole grains, legumes, nuts, seeds, and healthy (unsaturated) fats. Processed foods, added sugar, and refined grains were restricted. The diet was individually adjusted according to the participants’ medical backgrounds.

In addition, the registered dietitians recorded anthropometric measurements, administered nutritional (Food Frequency Questionnaire, FFQ), quality of life (The World Health Organization Quality of Life Brief Version, WHOQOL-BREF), and depression risk (GDS) questionnaires, and collected stool samples. Body mass index (BMI) was calculated according to body weight/(height)^2.

### Measurements

#### Questionnaires

The dietary assessment was performed using the 127-item FFQ, which was developed for the Israeli population. The development and validation process of this questionnaire is described in detail elsewhere (26). Briefly, the FFQ includes 127 food items with nine frequency options ranging from “never or less than once monthly” to “six or more times daily.” The questionnaire is semi-quantitative, and standard portion size is described for each food item. The portion-size estimates are based on information from the Israeli Ministry of Health. Participants are asked to report their average frequency of consumption during the past year.

The GDS questionnaire was used to screen for the risk of depression. A sum of points ranging from 0 to 4 indicates a normal to pre-depressed state, 5–8 points indicate mild depression, 9–11 points moderate depression, and 12–15 points severe depression (27).

The WHOQOL-BREF was used to evaluate the quality of life of the participants. It is composed of 26 items, divided into four domains: physical health, psychological well-being, social relationships, and environment. The physical health domain included seven items: pain/discomfort, energy/fatigue, sleep and rest, dependence on medication, mobility, activities of daily living, and working capacity. The psychological well-being domain included six items: feelings (positive and negative); self-esteem; thinking, learning and memory; concentration; body image; and spirituality, religion, and personal beliefs. The social relationships domain included three items: personal relationships, sexual activity, and practical social support. The environment domain included eight items: financial resources, information skills, recreation and leisure, home environment, access to health and social care, physical safety and security, physical environment, and transport. Two items of the WHOQOL-BREF are related to the general perception of quality-of-life and satisfaction with health (28).

#### Gut microbiota profiling

##### Fecal sampling

Participants received clear instructions for self-collecting fecal samples at their homes. The instructions included collecting samples during the morning hours, storing them in screw-capped collection containers filled with an RNase inhibitor solution (DNA/RNA Shield Fecal Collection Tube, Zymo Research, CA, USA), and keeping them at room temperature for a maximum of 1 week. The samples were transported at room temperature to the laboratory, and kept at −80°C until analysis.

##### Microbial DNA extraction and 16S rRNA amplicon sequencing

Microbial DNA was extracted using the Dneasy PowerSoil Kit (Qiagen, Hilden, Germany), according to the manufacturer’s instructions. DNA concentration and quality were assessed using the NanoDrop ND-1000 spectrophotometer (NanoDrop Technologies, Wilmington, DE, USA).

For library preparation, the V3-V4 hypervariable region of the 16S rRNA gene was amplified using the 341F and 785R primers with Illumina overhang adapter sequences, as previously reported (29). Amplicons were purified using Agencourt AMPure XP beads (Beckman Coulter, Brea, CA, USA), indexed with limited-cycle PCR using Nextera technology, and further cleaned up. The final libraries were sequenced on an Illumina MiSeq platform, with a 2 × 250 bp paired-end protocol according to the manufacturer’s instructions (Illumina, San Diego, CA, USA). Sequencing reads were deposited in the National Center for Biotechnology Information Sequence Read Archive (NCBI SRA; Submission SUB12494714).

##### Bioinformatics analysis

A mean 15,330 ± 3,758 sequences per sample were obtained. Raw sequences were processed according to a standardized pipeline based on PANDASeq (30) and QIIME 2 (31). After length and quality filtering, reads were grouped into amplicon sequence variants (ASVs) implementing DADA2 (32). Representative sequences were taxonomically assigned using a hybrid method involving VSEARCH (33) and a q2-classifier (34) trained on the Greengenes database (35). Alpha diversity was evaluated using the number of observed ASVs, and Shannon and Faith’s phylogenetic diversity metrics. Weighted and unweighted UniFrac distances were used to construct principal coordinate analysis plots.

#### SCFA extraction and analysis

##### SCFA extraction

SCFAs were extracted from 22 fecal samples (two from each of 11 participants, at T0 and T1) and analyzed as previously described (36). Briefly, each sample was thoroughly mixed using a vortex for 5 min. An aliquot of 0.5 mL of the mixed fecal solution was taken. Its pH was adjusted to 2–3 by adding 150 μL of orthophosphoric acid (16% v/v), after which it was kept at room temperature for 10 min with occasional shaking. The suspension was transferred into a polypropylene tube and centrifuged at 4°C for 5 min at 10,000 rpm. The supernatant was transferred to a chromatographic vial for gas chromatography analyses, and 2-methyl-butyric-acid (#109959, Sigma-Aldrich, USA) was added to each vial, to reach a final concentration of 0.001 M. To correct for injection variability between samples and minor changes in the instrument response, 2-methyl-butyric-acid was added as an internal standard. All the vials were stored at −20°C before gas chromatography mass spectrometry (GC–MS) analysis. The fecal sediment was dried at 60°C for 5–7 days, and its weight determined.

##### Gas chromatography mass spectrometry analysis

The GC–MS system consisted of an Agilent 7890A (Agilent Technologies, Palo Alto, CA, USA), equipped with an automatic liquid sampler (MPS2) (Gerstel, Mulheim, Germany) and coupled to an Agilent 5975C mass selective detector. Results were acquired using the Chemstation software (Hewlett–Packard, Palo Alto, CA, USA). The column used was a fused-silica capillary column with a free fatty acid phase (DB-FFAP 122–3232, J&W Scientific, Agilent Technologies Inc., USA) of 0.25 mm × 30 m × 0.25 μm. We used helium as the carrier gas at a flow rate of 60.453 mL/min. The initial oven temperature was 70°C, held for 0.75 min, raised to 160°C at 5°C/min, raised to 230°C at 20°C/min, and held for 5.0 min. A glass liner was used at the injection site to ensure maximum resolution and sensitivity of peaks. The injected sample volume for GC analysis was 1 μL, and the run time for each analysis was 27.25 min. The detector was operated in the selection ionization mode. Ion selection of the SCFAs was based on the retention time of standard compounds (WSFA-4, #47056, Sigma-Aldrich, St. Louis, USA).

### Statistical analysis

To assess whether the sample size was sufficient to detect differences in nutritional data, fecal microbiota composition and metabolites following the dietary intervention, a post-hoc power analysis was performed. Specifically, for each parameter measured during the intervention, i.e., nutrients, fecal metabolites, and relative abundances of taxa at family and genus level, a Wilcoxon rank-sum test with continuity correction was performed to assess the differences between pre- and post-intervention values. The effect size for each Wilcoxon test was estimated for each parameter from the W statistics as the square root of the squared W, divided by the sum of the squared W and the sample size prior to intervention. Power analysis was then performed using the pwr.2p2n.test function from the pwr R package, considering a significance level of 0.05. The median value of statistical power, calculated considering the power result for all parameters, was 92% (CI 95%: [0.87–0.99]).

We performed paired *t*-tests to compare nutritional data and SCFA concentrations before and after the intervention, using GraphPad Prism 9.0.0 (GraphPad Software, San Diego, California USA). A linear mixed model was estimated to identify influential variables in which the fixed effects were time, gender, BMI, and the four domains of the WHOQOL-BREF. The data were analyzed with the “lme4” package, using R, version 4.2.2 (37). For the gut microbiota, all statistical analyses were made using R, version 4.2.2 (37). Principal coordinate analysis plots were generated using the “vegan” (38) (REv2.6–4 package), and data separation was tested with the PERMANOVA test using the *adonis* function. Differences in microbiota composition and alpha diversity after the personalized diet were evaluated by Wilcoxon signed-rank tests. Box plots showing distributions were plotted using the “ggplot2” v3.4.0 R-package (39). Kendall correlations were sought between relative taxon abundances and nutritional data, and correlation plots were produced using the “ggcorrplot” package v0.1.4 (40). *P*-values were corrected for multiple comparisons using the Benjamini-Hochberg method. A false discovery rate ≤ 0.05 was considered statistically significant. A *p*-value ≤0.1 was considered as a trend.

## Results

### Description of the study cohort

**Figure 1:**
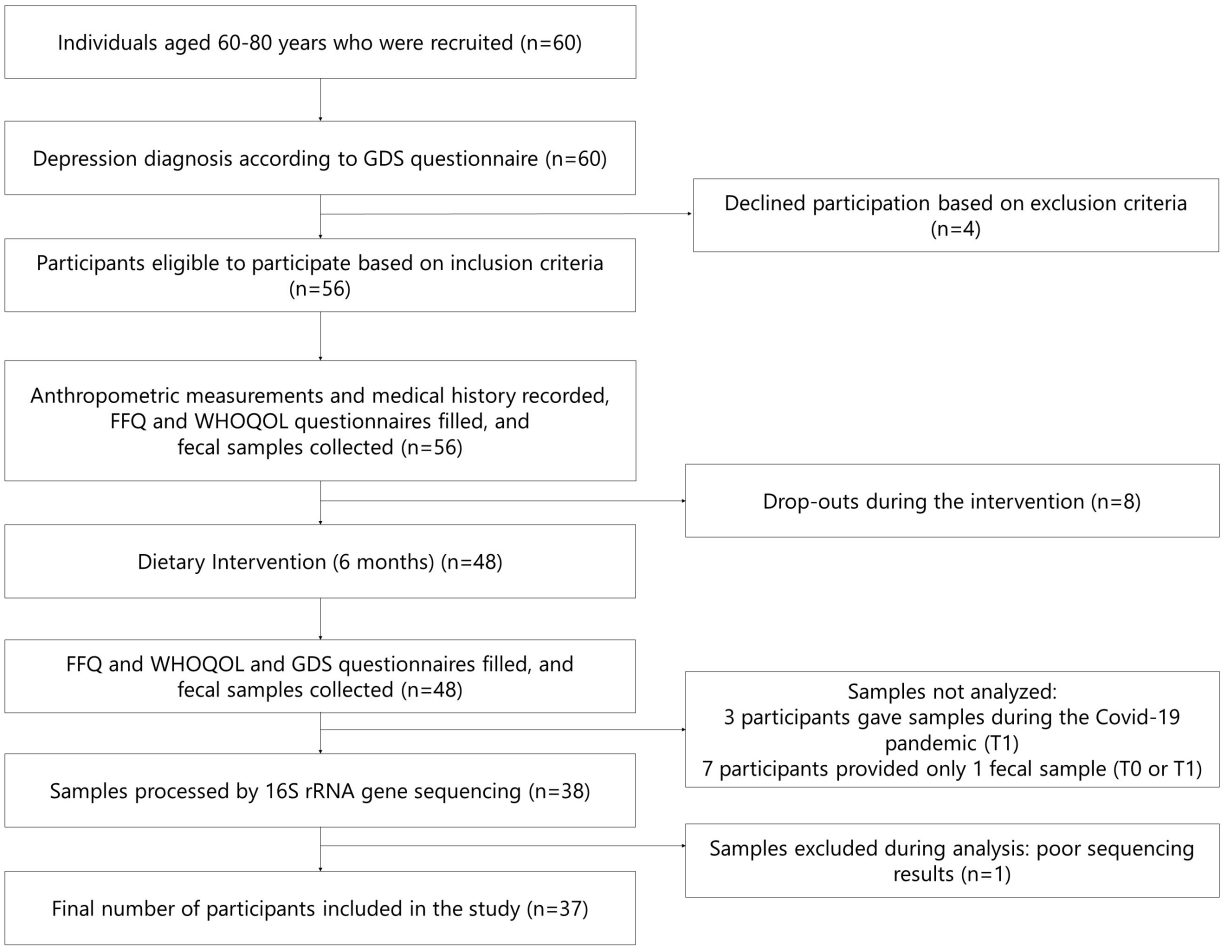
Flowchart of the study design.

Sixty individuals responded to the announcements that recruited participants for the study. Of them, 56 met the study eligibility criteria. During the dietary intervention, eight volunteers ended their participation. The reasons for participant dropouts during the intervention were loss of interest or motivation, claimed to be lack of perceived benefit, dissatisfaction with the study procedures, or personal reasons unrelated to the research. Forty-eight people finished the intervention, and 37 were included in this study. Ten individuals had to be excluded during the analysis of the results since three gave their samples during the COVID-19 pandemic, a potential confound, and 7 participants provided only one fecal sample for analysis (baseline or after 6 months of intervention). One individual was excluded due to poor sequencing results of the fecal sample. Figure 1 shows a flowchart of the study.

The mean age of the 37 participants was 69 ± 5 years; 10 were male and 27 were female. Table 1 summarizes the means and standard deviations for all the measured parameters before and after 6 months of nutritional intervention. The raw data can be found in Supplementary Table S1.

**Table 1:** BMI and questionnaire data of the study participants at baseline (T0) and after intervention (T1).

Parameter	T0	T1
	Means and standard deviations	Mean and standard deviations
BMI (kg/m^2^)	28.2 ± 5.30	28.1 ± 5.07
GDS	3.43 ± 2.39	2.30 ± 1.97
Domains of the World Health Organization Quality of Life questionnaire		
Physical health	68.5 ± 11.2	69.8 ± 10.2
Psychological well-being	61.7 ± 11.6	64.3 ± 12.2
Social relationships	66.1 ± 19.9	69.7 ± 19.0
Environment	72.0 ± 10.0	75.3 ± 10.1

BMI, body mass index; GDS, Geriatric Depression Scale; T0, Baseline; T1, After 6 months of intervention.

### Changes in depression and quality of life following the personalized diet intervention

We used a mix model to examine changes in depression and quality of life following 6 months on the personalized diet. According to the likelihood ratio test (Table 2), the model was statistically significant (*X*^2^ (9) = 36.0, *p*-value <0.001, Akaike information criterion = 336.7). GDS levels were significantly lower after the intervention, in an analysis that controlled for age, gender, BMI, and WHOQOL-BREF domains. The psychological domain of the WHOQOL-BREF questionnaire was significantly associated with lower GDS values (*p*-value <0.05).

**Table 2:** Results of a likelihood test of predicting Geriatric Depression Scale values by gender, intervention time, BMI, age, and domains of the World Health Organization Quality of Life Brief Version.

Predictor	Estimate	Standard error	df	*t*-value	*p*-value
Gender	−1.23	0.544	45.1	−2.27	**0.0284**
T1	−0.912	0.419	31.6	−2.18	**0.0369**
BMI	0.153	0.236	50.1	0.647	0.520
Age	−0.302	0.228	43.1	−1.33	0.192
Domains of the World Health Organization Quality of Life questionnaire					
Physical health	0.270	0.240	55.6	−1.13	0.266
Psychological well-being	−0.742	0.274	58.9	−2.71	**0.00875**
Social relationships	−0.374	0.227	61.8	−1.65	0.105
Environment	0.237	0.229	62.7	1.03	0.305

According to the likelihood ratio test, the model was statistically significant (X^2^(9) = 34.0, *p*-value < 0.001, Akaike information criterion = 336.7). T1, intervention time of 6 months; BMI, body mass index; df, degrees of freedom. The bold numbers refer to statistically significant results (*p*-value < 0.05).

### Changes in macro- and micronutrient consumption following the personalized diet intervention

Nutritional facts of the participants were collected using the FFQ at T0 and T1. The energy consumption of the participants did not change significantly before and after intervention (*p*-value = 0.498). Furthermore, percentages of fat, protein and carbohydrates did not differ between T0 and T1 (*p*-value = 0.159, 0.355, and 0.081, respectively). The raw data can be found in Supplementary Table S1. We performed a ratio paired *t*-test to examine a set of nutrients that would be expected to increase with adherence to a Mediterranean diet. The distribution of the ratio of measurements was normal according to the Shapiro–Wilk test (*p*-value = 0.587 and *p*-value = 0.214 for T0 and T1, respectively). As shown in Table 3, the ratio of folate, lutein and zeaxanthin were significantly higher after the intervention. In contrary, EPA, and DHA intake was significantly lower after the intervention.

**Table 3:** Mediterranean diet nutrients of the participants at baseline (T0) and after the intervention (T1).

Nutrient (units)	Mean of log(T0)	Mean of log(T1)	Geometric mean of ratios	SEM of log(ratios)	*t* ratio	*P*-value
Lutein and zeaxanthin (mcg)	3.40	3.45	0.890	0.0219	2.32	**0.0263**
Fiber (g)	1.51	1.52	0.988	0.0206	0.251	0.803
Vitamin E (mg)	1.08	1.10	0.950	0.0171	1.30	0.201
Vitamin B6 (mg)	0.385	0.397	0.971	0.0188	0.670	0.507
Folate (mcg)	2.53	2.57	0.915	0.0174	2.22	**0.0329**
MUFA (g)	1.48	1.52	0.922	0.0231	1.53	0.134
Oleic acid (g)	1.44	1.48	0.920	0.0232	1.56	0.129
PUFA (g)	1.34	1.38	0.914	0.0222	1.75	0.0884
EPA (g)	−1.43	−1.31	0.751	0.0591	2.11	**0.0423**
DHA (g)	−0.976	−0.873	0.788	0.0462	2.24	**0.0316**
Iron (mg)	1.11	1.13	0.961	0.0184	0.946	0.350
Zinc (mg)	1.05	1.06	0.968	0.0160	0.891	0.379
Selenium (mcg)	2.06	2.06	0.989	0.0178	0.272	0.787

SD, standard deviation; g, grams; mg, milligrams; mcg, micrograms; MUFA, monosaturated fatty acids; PUFA, polyunsaturated fatty acids; EPA, eicosapentaenoic acid; DHA, docosahexaenoic acid; SEM, standard error of means. The bold numbers refer to statistically significant results with *p*-value < 0.05.

### Changes in SCFA concentrations of fecal samples following the personalized diet intervention

**Figure 2:**
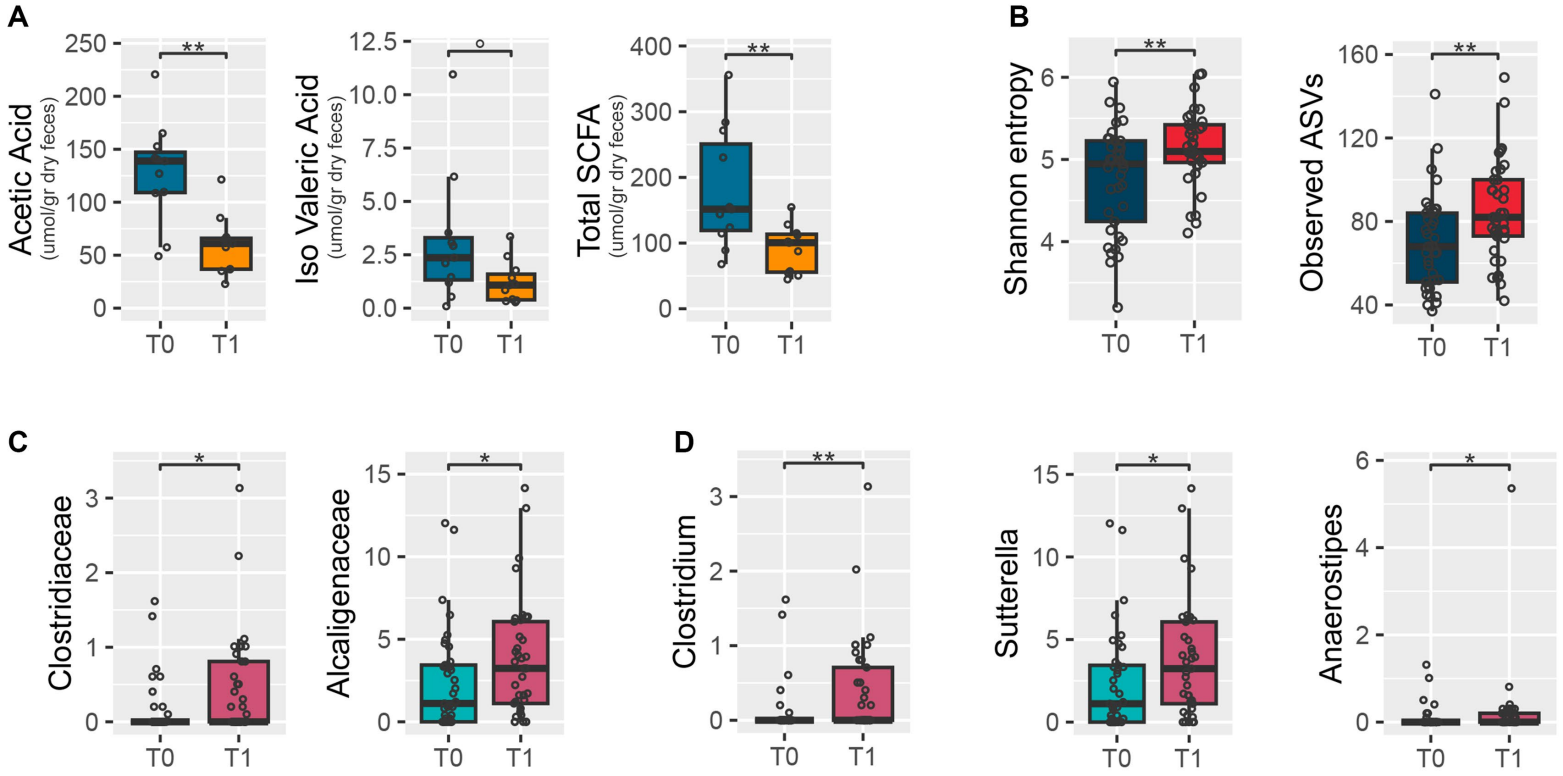
Fecal short-chain fatty acid concentrations and microbiota at baseline (T0) and after the intervention (T1). The box plots show distributions of short-chain fatty acid concentrations **(A)**, alpha diversity of the microbiome measured with Shannon’s entropy (left) and the number of observed amplicon sequence variants (right) **(B)**, and relative abundances of bacterial families **(C)** and genera **(D)** differentially represented at T0 and after T1. Wilcoxon signed-rank test: **p-value<0.01; *p-value<0.05; 0*p*-value<0.1.

Fecal SCFA concentrations were determined for 11 participants at T0 and T1. As shown in Figure 2A, concentrations of acetic acid and total SCFAs were significantly lower after the intervention (*p*-values = 0.0005 and 0.007, respectively), while isovaleric acid showed a decreasing trend.

### Changes in the gut microbiota following the personalized diet intervention

According to the number of observed ASVs and Shannon’s entropy, the dietary intervention resulted in significantly increased values of alpha diversity (Wilcoxon test, *p*-value = 0.006 and 0.01, respectively) (Figure 2B). Yet, no significant change was observed in beta diversity between time points (PERMANOVA, *p*-value >0.9) (Supplementary Figure 1). The relative abundance of the families *Clostridiaceae* and *Alcaligenaceae* (*p*-value = 0.021 and 0.042, respectively) (Figure 2C), and specifically of the genera *Clostridium*, *Sutterella*, and *Anaerostipes* (*p*-values = 0.009, 0.044, and 0.049, respectively) (Figure 2D), increased at T1 compared to T0.

### Interrelations between food nutrients, gut microbiota, and mental health

Fecal samples were categorized according to scores on the psychological well-being domain on the WHOQOL-BREF, and to scores on the GDS. Briefly, the scores were converted into the following categories: (i) 25–50, 51–75, and 76–100 for the WHOQOL-BREF psychological well-being domain (there were no scores below 25); and (ii) no depression and pre-depression (GDS ≤ 4), and depression (GDS > 4) for the GDS. These categories were used to stratify the fecal samples to examine differences between them during the intervention. At both T0 and T1, no significant segregation in gut microbiota structure was found according to WHOQOL-BREF and GDS categories, based on PERMANOVA testing on weighted and unweighted UniFrac distances of beta diversity (Supplementary Figure 2).

**Figure 3:**
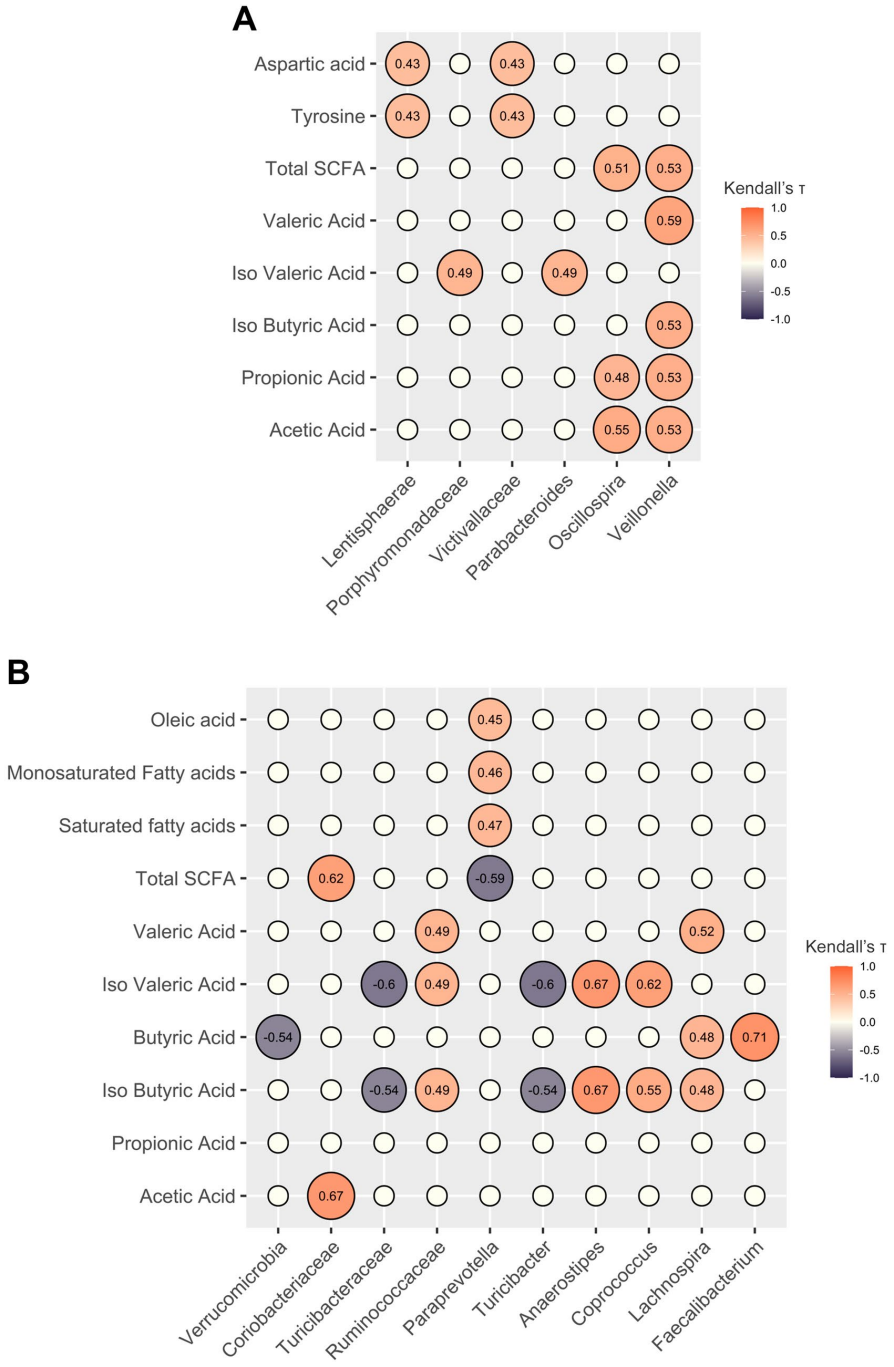
Correlations between gut microbiota components and host metadata. Correlation plots showing only significant associations (false discovery rate-corrected Kendall *p*-value <0.05) of relative abundances of gut microbiota components with nutritional data and SCFA levels before **(A)** and after **(B)** the personalized diet intervention. The empty circles represent non-significant correlations. The numbers within the circles represent Kendall’s tau coefficients.

Before the intervention, total fecal SCFA concentrations, particularly acetic acid and propionic acid, strongly correlated with the genera *Oscillospira* and *Veillonella*. At T0, *Veillonella* correlated with isobutyric and valeric acids as well, while isovaleric acid correlated with the genus *Parabacteroides* and the family *Porphyromonadaceae*. Regarding the nutritional data, the families *Victivallaceae* and *Lentisphaerae* correlated with tyrosine and aspartic acid. After the intervention, correlations were detected between taxa and SCFA concentrations, yet the acids involved were different. Specifically, isovaleric and isobutyric acids correlated positively with the genera *Anaerostipes* and *Coprococcus*, and negatively with *Turicibacter*; and *Lachnospira* correlated positively with isobutyric, butyric, and valeric acids. Lastly, *Faecalibacterium* correlated strongly with butyric acid levels. Total SCFA levels correlated negatively with the genus *Paraprevotella*, which in turn correlated positively with levels of oleic acid, monosaturated fatty acids, and saturated fatty acids (Figure 3).

## Discussion

We performed a Mediterranean-based personalized diet intervention based on participants’ medical, nutritional, and anthropometric information and individual preferences, barriers, behaviors, and objectives. The main finding of this study is a reduction in depression symptoms in older adults after 6 months of intervention compared to baseline. This reduction was associated with an increase in quality of life. In addition, after the intervention, dietary intake of folate, lutein, zeaxanthin, EPA, and DHA was significantly changed.

Although most of the available evidence in support of personalized nutrition comes from observational studies, there is increasing evidence that a tailored diet is more effective than a conventional one-size-fits-all approach (41, 42). When based on the Mediterranean guidelines, personalized interventions are expected to benefit mental health, as recent literature suggests that the Mediterranean diet may help to prevent depressive illnesses and potentially treat depression and anxiety (43, 44). Indeed, we show reduced depression symptoms and improved quality of life in older adults after 6 months of a Mediterranean-based personalized diet intervention. Although bi-weekly dietitian visits may have contributed to the positive findings, we observed changes in micronutrient intake and microbiome composition, strengthening our hypothesis that tailored diets may play a role in reducing depression symptoms and improving quality of life in older adults.

Studies suggest that adhering to a varied diet rich in vegetables, fruit, nuts, fish, and olive oil, characteristic of the Mediterranean diet, may offer protection against depression (45). Lutein and zeaxanthin are dietary carotenoids commonly found in fruits and vegetables like spinach, kale, broccoli, peas, parsley, and yellow corn. These carotenoids accumulate in significant concentrations in the human brain, promoting brain health, preventing cognitive decline (46), and potentially influencing mood and mental well-being. Research indicates that certain gut bacteria have the ability to metabolize these carotenoids into various beneficial metabolites, which may have enhanced bioavailability and antioxidant properties (47). Moreover, studies have shown that lutein and zeaxanthin can impact the composition and increase the diversity of the gut microbiota (48). After implementing our Mediterranean-based personalized intervention, we observed a significant and positive increase in the consumption of the carotenoids lutein and zeaxanthin, as well as an improvement in gut microbiota diversity. These changes may potentially contribute to reducing depression symptoms and improving overall quality of life.

Following the Mediterranean-based personalized intervention, we also observed an increase in folate intake. Folate is a water-soluble B vitamin that is abundant in the Mediterranean diet and serves as a critical cofactor in various biochemical pathways, including DNA synthesis and methylation (49). Moreover, folate plays a crucial role in neurotransmitter synthesis, particularly in the production of serotonin, dopamine, and norepinephrine, which are essential for mood regulation and emotional stability (50). Its metabolism and bioavailability have been suggested to influence gut-brain signaling pathways, with potential implications for mental health (51). Previous studies and a meta-analysis (52–54) have reported significant correlations between folic acid levels and depression. Low blood levels of folic acid were associated with an increased risk of depression, while a high dietary intake of folic acid appeared to have a protective effect against depression. Our findings may further support the notion of the importance of folate intake for good mental health.

The three primary omega-3 fatty acids are alpha-linolenic acid (ALA), EPA and DHA. ALA is found mainly in plant oils such as flaxseed, soybean, and canola oils. DHA and EPA are found in fish and other seafood, cannot be synthesized by the human body, and must be directly supplied by the diet or converted from ingested ALA. EPA and DHA are essential nutrients for brain development and health, as they play a pivotal role in the regulation of synaptic plasticity, neurogenesis (55), dopaminergic and serotonergic neurotransmission (56), and HPA axis activity (57). Both have been shown to exert significant effects on mental health, such as mood, cognitive functioning, anxiety, and depression, and modulation of gut microbiota composition (58). Although omega-3 fatty acid consumption is highly recommended as part of the Mediterranean diet (59), we observed significantly lower EPA and DHA intake after the intervention. This result may be due to the participants’ lower intake of fish and seafood during the intervention. The levels of ALA and total unsaturated fatty acids did not differ at T1.

Increased diversity of the gut microbiota was observed after the intervention. Such an increase is generally considered to be a hallmark of a healthier microbial community and an overall healthier organism (60). Some compositional changes were also found, although none of them were significantly linked to scores on the WHOQOL-BREF. Nevertheless, it should be noted that the intervention resulted in increased proportions of *Sutterella* and *Anaerostipes*, both of which have been reported to be underrepresented in individuals with depressive disorders (61). Notably, *Anaerostipes* has been shown to ameliorate depressive-like behaviors in mouse models, probably through butyrate-mediated mechanisms related to serotonergic system balance and trophic factor expression (62). Furthermore, *Anaerostipes* belongs to the *Lachnospiraceae* family, which has been associated with higher carotenoid concentrations (48), suggesting a possible link with the increase in lutein and zeaxanthin.

Prior to the intervention, significant correlations were observed between total fecal SCFA concentrations, specifically acetic acid and propionic acid, and the genera *Oscillospira* and *Veillonella*. Additionally, the genus *Parabacteroides* showed a positive association with isovaleric acid. These findings are consistent with existing research, which identifies *Oscillospira* and *Veillonella* as abundant gut bacteria known for their SCFA production (63, 64), while *Parabacteroides* is known to ferment proteins and produce isovaleric acid (65). Following the intervention, *Lachnospira* and *Fecalibacterium* showed positive correlations with SCFAs, particularly with butyric acid, further emphasizing the contribution of these genera as SCFA producers (66).

The total SCFA concentration, as well as the concentrations of acetic and isovaleric acid, were lower following the intervention. Again, although these are only associations, fecal levels of isovaleric acid have been shown to correlate with depression by interfering with synaptic neurotransmitter release (67). SCFAs are typically derived from microbiota fermentation of fiber, while branched-chain fatty acids (such as isovaleric acid) are derived from microbial fermentation of branched-chain amino acids (68). However, no changes in fiber or protein intake were found. Furthermore, based on the available literature, the taxa identified as altered after the intervention cannot be easily linked to the changes in metabolites. This may suggest that other components of the diet and/or specific syntrophic networks (yet to be elucidated) may be involved in microbiota-related metabolic outputs.

This study has several limitations. First, the assessments of depression, quality of life, and food frequency were based on questionnaires, which are prone to individual-response bias. FFQs are widely used in nutritional research to assess dietary intake over time. However, they have several limitations, mainly inaccuracy in special populations and food composition databases, memory and recall bias and lack of short-term variation, portion size estimation and social desirability bias (69, 70). We implemented various strategies to overcome or mitigate these challenges, such as the use of a validated and tailored FFQ developed for the Israeli population, constantly updating the database of food items. Bi-weekly visits by the clinical dietitians included 24-h dietary recalls, in combination with the FFQ, to capture short-term variations and cross-validate the data. In addition, photographs were used to help participants estimate portion sizes and improve accuracy in reporting food consumption. Second, the study lacks a control group. Although we explained why such a group was not used in this study, we recognize the need for a control group to be able to draw clearer conclusions about the effects of the intervention on depression symptoms and quality of life. Third, profiling the fecal microbiome using 16S rRNA amplicon sequencing did not allow identification of microbial taxa in depth (i.e., at high taxonomic resolution) and breadth (our data are limited to bacteria), nor their functions. Finally, fecal analysis has limitations, given that microbiome profiles, and metabolite concentrations, such as SCFAs, differ along the intestinal tract.

Overall, our study found that older adults who underwent the personalized diet intervention experienced a reduction in depression symptoms, an improvement in quality of life compared to their baseline measurements and changes in nutrient intake. The positive changes in mood were associated with increased consumption of certain dietary components related to brain function and mood regulation. The intervention also led to an increase in gut microbiota diversity, which is generally considered to be a sign of a healthier microbial community. Although specific compositional changes were observed, they were not significantly correlated with quality-of-life scores. Further investigation is required to elucidate the underlying mechanisms connecting food and mental well-being, and to ascertain the specific ways and optimal timing in which nutrition can be utilized to improve mental health.

## Data availability statement

Sequencing reads were deposited in the National Center for Biotechnology Information Sequence Read Archive (NCBI SRA), accession number PRJA991320.

## Ethics statement

The studies involving humans were approved by the Ethics Committee of the Meir Medical Center (approval no 0186-16-COM1). The studies were conducted in accordance with the local legislation and institutional requirements. The participants provided their written informed consent to participate in this study.

## Author contributions

FM wrote the first draft of the manuscript, conceived, and coordinated the work. STu analyzed the results and reviewed the manuscript. MF organized the metadata and performed the biostatistical analysis. MB wrote sections of the manuscript and performed the microbiome analysis. AV contributed to the design of the study. AO provided ethical approve. STa contributed to conception, funding acquisition, and design of the study. All authors contributed to manuscript revision, read, and approved the submitted version.

## Funding

This research received funding from the Israeli Ministry of Science and Technology (grant number: 312896). The funder was not involved in the study design, data collection, analysis of data, manuscript drafting, or submission.

## Conflict of interest

The authors declare that the research was conducted in the absence of any commercial or financial relationships that could be construed as a potential conflict of interest.

## Publisher’s note

All claims expressed in this article are solely those of the authors and do not necessarily represent those of their affiliated organizations, or those of the publisher, the editors and the reviewers. Any product that may be evaluated in this article, or claim that may be made by its manufacturer, is not guaranteed or endorsed by the publisher.
