# Supplementary material for: A personalized diet intervention improves depression symptoms and changes microbiota and metabolite profiles among community-dwelling older adults
Source: Front Nutr. 2023 Sep 14;10:1234549. doi: 10.3389/fnut.2023.1234549 (PMC10547149; doi:10.3389/fnut.2023.1234549)
Supplement: Supplementary file 1 [file Data_Sheet_1.PDF]

Supplementary Material

Supplementary Material

A personalized diet intervention improves depression symptoms and changes microbiota and metabolite profiles among community-dwelling older adults

Faiga Magzal*, Silvia Turroni, Marco Fabbrini, Monica Barone, Adi Vitman Schorr, Ariela Ofran, Snait Tamir

*** Correspondence:** Faiga Magzal: faigam@migal.org.il

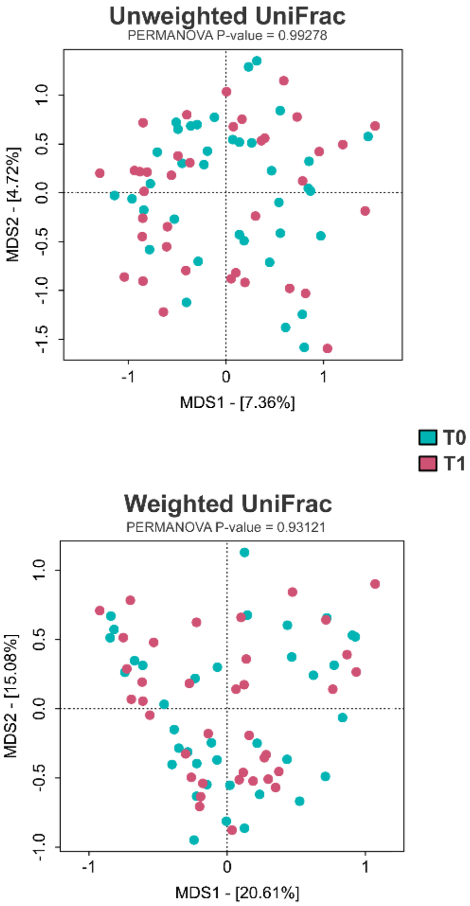

Supplementary Figure 1: Beta diversity of the gut microbiota before and after the personalized diet. Principal coordinate analysis plots representing unweighted and weighted UniFrac distances between the gut microbial profiles of the study participants, before (T0) and after (T1) the intervention. No significant segregation was found.

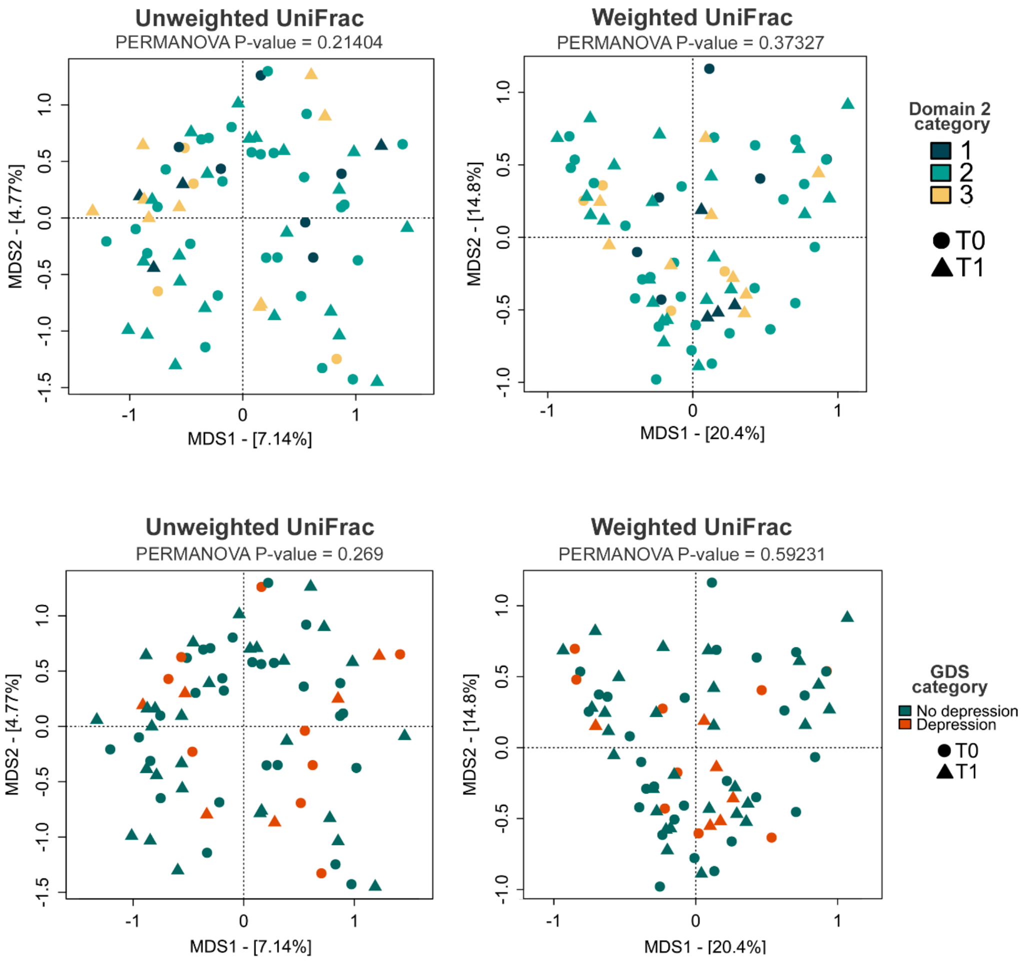

Supplementary Figure 2: Beta diversity measures in relation to categories of Domain 2 (psychological well-being) of the World Health Organization Quality of Life Brief Version and of the Geriatric Depression Scale. Weighted and unweighted UniFrac distances were used to compute principal coordinate analysis plots. Points were colored according to categories of the psychological well-being domain of the World Health Organization Quality of Life Brief Version (upper plots), and categories of the Geriatric Depression Scale GDS (bottom plots). Circles refer to baseline values (T0). Triangles refer to values at 6 months after the intervention (T1). No significant segregation was found between the time points.
